# Neuronal expression of muskelin in the rodent central nervous system

**DOI:** 10.1186/1471-2202-8-28

**Published:** 2007-05-02

**Authors:** Nadia Tagnaouti, Sven Loebrich, Frank Heisler, Yvonne Pechmann, Susanne Fehr, Adèle De Arcangelis, Elisabeth Georges-Labouesse, Josephine C Adams, Matthias Kneussel

**Affiliations:** 1 Zentrum für Molekulare Neurobiologie Hamburg, ZMNH, Universität Hamburg, Germany; 2 Institut de Génétique et de Biologie Moléculaire et Cellulaire, IGBMC, CNRS/INSERM/ULP, Parc d'Innovation, Illkirch, Strasbourg, France; 3 Department of Cell Biology, Lerner Research Institute, Cleveland Clinic Foundation, 9500 Euclid Avenue, Cleveland, OH 44195, USA

## Abstract

**Background:**

The kelch repeat protein muskelin mediates cytoskeletal responses to the extracellular matrix protein thrombospondin 1, (TSP1), that is known to promote synaptogenesis in the central nervous system (CNS). Muskelin displays intracellular localization and affects cytoskeletal organization in adherent cells. Muskelin is expressed in adult brain and has been reported to bind the Cdk5 activator p39, which also facilitates the formation of functional synapses. Since little is known about muskelin in neuronal tissues, we here analysed the tissue distribution of muskelin in rodent brain and analysed its subcellular localization using cultured neurons from multiple life stages.

**Results:**

Our data show that muskelin transcripts and polypeptides are expressed throughout the central nervous system with significantly high levels in hippocampus and cerebellum, a finding that resembles the tissue distribution of p39. At the subcellular level, muskelin is found in the soma, in neurite projections and the nucleus with a punctate distribution in both axons and dendrites. Immunostaining and synaptosome preparations identify partial localization of muskelin at synaptic sites. Differential centrifugation further reveals muskelin in membrane-enriched, rather than cytosolic fractions.

**Conclusion:**

Our results suggest that muskelin represents a multifunctional protein associated with membranes and/or large protein complexes in most neurons of the central nervous system. These data are in conclusion with distinct roles of muskelin's functional interaction partners.

## Background

Muskelin was originally identified as a molecule required in cellular responses to the extracellular matrix (ECM) component thrombospondin-1 (TSP-1) [1]. Muskelin overexpression promotes cell attachment to the C-terminus of TSP-1 and antisense depletion of muskelin expression leads to reduced cell attachment, cell spreading and cytoskeletal reorganization [1]. TSP-1 is a member of the thrombospondin (TSP) family of widely-expressed, multifunctional ECM proteins [2,3]. TSPs secreted by immature astrocytes during embryonic development promote central nervous system (CNS) synaptogenesis [4]. Muskelin transcripts are expressed in different tissues of developing mouse embryos [5] and Northern blot analysis as well as RT-PCR detected muskelin transcripts in many adult tissues including brain [1,6], however whether the synaptogenic effect of TSPs involves muskelin function is currently unclear.

A reported direct binding partner of muskelin is the cyclin-dependent kinase 5 (Cdk5) activator, p39, that is abundant in the CNS [7], displays highest expression in cerebellum and hippocampus, and partially localizes to synaptophysin-positive synapses [8]. In COS cells and lens epithelial cells, coexpression of p39 and muskelin recruits intracellular muskelin toward the cell periphery [6], however whether muskelin also localizes at synaptic sites in neurons is presently unknown. Notably, overexpression of Cdk5 and p39 resulted in significantly higher rates of synapse formation in a neuroblastoma cell/myotube co-culture system [8], and both the knockout of Cdk5 [9] as well as the double-knockout of p39 and its homologue p35 in mice [10] lead to widespread disruption of neuronal migration and brain development. Together, these data indicate a critical role for both TSPs and Cdk5/p39 signalling pathways in synapse formation and suggest the hypothesis that muskelin, reported to interact functionally with both systems, might also play a role in synaptogenic mechanisms.

**Figure 1:**
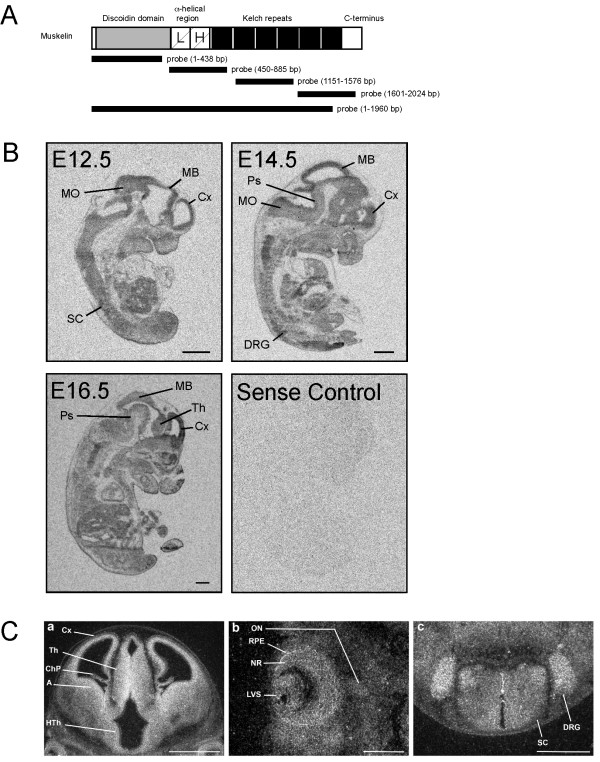
*In situ *hybridization analysis of muskelin transcripts in the developing mouse embryo. (A) Schematic representation of muskelin. Individual protein domains are indicated. L: LisH domain; H: LisH homology domain (also known as CTLH for: C-terminal to LisH domain). The probes used for *in situ *hybridization are indicated with black bars. (B) Micrographs of X-ray films showing whole embryo sagittal sections from embryonic stages E12.5, E14.5 and E16.5. Data obtained with probe 1 are shown in representation for similar results with probes 2–4. The sense control displays a parallel experiment using sense RNAs as probe. (C) E12.5 coronal section through a posterior region at the midbrain level using probe 5 (a). High expression levels of muskelin mRNA were observed in the subventricular zone of the cortex, and the amygdala as well as in the neuroepithelium of the thalamus and hypothalamus. High magnification of a coronal section through the eye (b) at E12.5 showing the presence of muskelin transcripts in the lens vesicle, the neural retina and the retinal pigmented epithelium. Weak signal was detected in the optic nerve. (c) Coronal section through the spinal cord, showing muskelin mRNA expression in the ependymal and ventral mantle layers and in the DRG. Key: A, amygdala; ChP, choroid plexus; Cx, cortex; DRG, dorsal root ganglia; HTh, hypothalamus; LVS, lens vesicle; MB, midbrain; MO, medulla oblongata; NR, neural retina; ON, optic nerve; Ps, pons; RPE, retinal pigmented epithelium; SC, spinal cord; Th, thalamus. Scale bars: (B) 1 mm each, (C) (a) 1 mm, (b) and (c) 500 nm.

Other reported interaction partners of muskelin include the prostaglandin EP3 receptor [11] and a protein complex consisting of Twa 1 and RanBPM [12]. At the molecular level, muskelin is a multidomain protein that contains an amino-terminal discoidin domain, an α-helical, Lissencephaly-1 homology (LisH) motif and a C-terminal to LisH (CTLH) motif (Figure 1A). The C-terminal half of muskelin contains six repeated kelch motifs [13]. Each kelch repeat forms a four-stranded antiparallel beta-sheet that corresponds to a blade in a beta-propeller structure. Whereas some kelch-repeat proteins bind to actin, others have unique binding partners [14]. Muskelin does not directly interact with actin *in vitro *[13] and although myc-tagged muskelin located at actin-rich plasma membrane regions in lens epithelial cells [6], muskelin has only weak colocalization with actin microfilaments in mouse skeletal myoblasts, smooth muscle cells and COS-7 cells [13]. Similar to other kelch repeat proteins, that are known to assemble into dimers or oligomers, muskelin self-associates through a head-to-tail mechanism [13], features which might be important for the proposed functions of muskelin in the reorganization of cytoskeletal elements [1].

Our study provides the first comprehensive spatio-temporal analysis of muskelin expression in neuronal tissues and at the subcellular level in neurons.

## Results

### Distribution of muskelin transcripts in the rodent central nervous system

**Figure 2:**
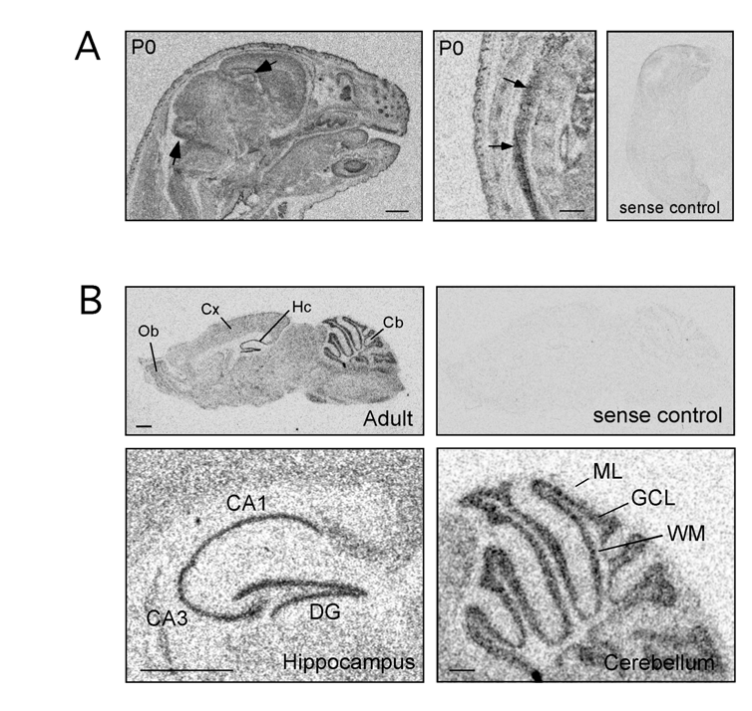
*In situ *hybridization analysis of muskelin transcripts in newborn (P0) and adult stage (P42). (A) Postnatal stage P0. Left: basal transcript expression is found throughout the brain with signals in the hippocampal formation and the cerebellum above average (arrows). Middle: at the newborn stage transcripts are also prominent in the spinal cord (arrows). Right: The sense control displays a parallel experiment using sense RNAs as probe. (B) Adult stage. Basal muskelin transcript expression is found throughout the brain. Hybridization signals in cerebellum and hippocampus are particularly strong, as compared to other brain regions. The sense control displays a parallel experiment using sense RNAs as probe. In the hippocampus, muskelin transcripts display highest concentration in cell bodies of pyramidal cells in CA1 and CA3 regions, as well as in cell bodies of dentate gyrus granule cells. In the cerebellum, muskelin transcripts are mainly found in cell bodies of granule and Purkinje cells. Key: CA1, Hippocampal CA1 region; CA3, Hippocampal CA3 region; Cb, Cerebellum; Cx, Cortex; DG, Dentate gyrus; GCL, Granule cell layer; Hc, Hippocampus; ML, Molecular layer; Ob, Olfactory Bulb; WM, White matter. Scale bars: (A) and (B) 1 mm.

To analyze muskelin mRNA distribution in the developing mouse central nervous system (CNS), we used four independent radioactive probes corresponding to sequences that encoded non-overlapping muskelin sequences (1–438 bp; 450–885 bp; 1151–1576 bp; 1601–2024 bp) (Figure 1B and 2) or all domains except the C-terminus (1–1960 bp) (Figure 1C). At embryonic stage E12.5, prominent hybridization signals indicative of muskelin mRNA transcripts were detected in the developing CNS (Figure 1B). At E12.5, high levels of muskelin expression were observed in the neuroepithelium of the cortex, hippocampus, amygdala, and in the thalamus and hypothalamus as shown in high magnification coronal view (Figure 1C a). Additional high expression was observed in the trigeminal ganglia (data not shown) and dorsal root ganglia (Figure 1C c). Lower levels were also seen in the eye (neural retina and lens vesicle, retinal pigmented epithelium) (Figure 1C b), and in the spinal cord (Figure 1C c). Specificity of *in situ *hybridization was controlled by using alternative probes corresponding to different portions of the muskelin coding sequence and by application of sense RNA probes (Figures 1, 2 and data not shown). At embryonic stages E14.5 and E16.5, mRNA was also detectable in the developing cortex, the midbrain, the pons-midbrain region as well as the medulla oblongata and the dorsal root ganglia (Figure 1B).

We then performed *in situ *hybridization around birth using P0 tissue. Here, the analyses revealed that muskelin mRNA transcripts were present in all brain regions, however transcript levels were above average in the hippocampal formation and cerebellum of newborn animals (arrows). Hybridization signals were clearly detectable in the spinal cord at this stage (Figure 2A). Thus, our data indicate that muskelin transcripts are abundant in the second half of mouse embryonic development with a widespread distribution throughout the CNS.

Muskelin has been identified to mediate intracellular responses to thrombospondin-1, however thrombospondins-1 and -2, both identified as synaptogenic proteins, are expressed in the embryonic [15] and postnatal brain but become downregulated in the adult CNS [4]. In contrast, thrombospondin-4 (TSP-4) is highly expressed in the adult CNS [16]. We therefore also analyzed muskelin transcripts at adult stages and performed *in situ *hybridization on tissue derived from P42 mice. Muskelin transcripts were prominent throughout the brain and notably displayed significantly strong signals in the adult hippocampal formation and the adult cerebellum (Figure 2B). These data suggest that muskelin might play additional, as yet unidentified, roles in neurons together with other thrombospondins or unrelated proteins.

### Distribution of muskelin protein in the adult brain

**Figure 3:**
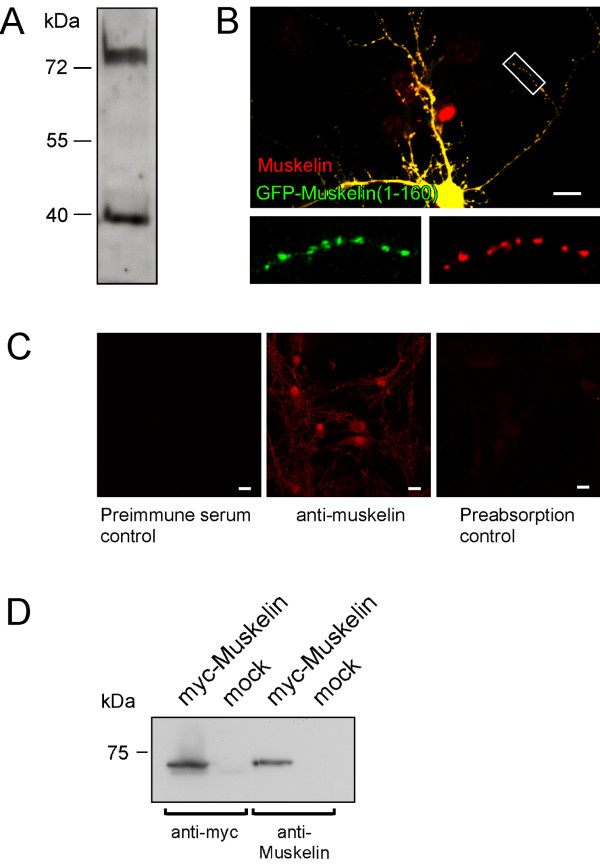
Characterization of a newly generated muskelin-specific antibody. (A) Western blot analysis of cell lysate from adult hippocampus. The antibody detects full-length muskelin and a previously reported putative proteolytic fragment. (B) Immunostaining of cultured hippocampal neurons expressing GFP-Muskelin (1–160) (green channel). The muskelin-specific antibody (red channel), originally generated against N-terminal muskelin sequences, recognizes the overexpressed green fluorescent fusion protein, as revealed from the precise colocalization of green and red puncta in distal dendrites (yellow). The red nucleus in the middle of the image represents immunoreactivity of an untransfected neuron close to the analysed cell in a different optical layer. The boxed region is shown as individual channels. Scale bar: 15 μm. (C) Preabsorption control upon antibody clearance with GST-Muskelin 1–280. Neither the preimmuneserum control (left), nor the preabsorption control (right) produce unspecific signals. In contrast, immunostaining with anti-muskelin (middle) reveals distinct immunoreactivity patterns. Scale bar: 15 μm. (D) Analysis of HEK293 cell extracts upon expression of myc-muskelin or empty vector (mock) with either myc- or muskelin-specific antibody. The newly generated antibody against muskelin recognizes a band of the same size, as detected with anti-myc.

We next raised a muskelin-specific antibody against the muskelin N-terminus (residues 1–280) to analyze whether the observed mRNA distribution corresponds to the protein distribution pattern of muskelin in brain. Western blot analysis revealed that the newly generated antibody detects both full length muskelin [1,6] and a previously reported N-terminal muskelin fragment of about 40 kDa that presumably represents a proteolytic fragment [1] (Figure 3A). Accordingly, an additional independent muskelin-specific antibody raised in our laboratory, detects both corresponding bands in Western blots (data not shown). As a further control, we expressed a GFP-fusion protein harbouring the N-terminal residues 1–160 in cultured hippocampal neurons. This polypeptide includes the muskelin residues used for antibody production. Indeed, our antibody recognized GFP-Muskelin (1–160) with high specificity, as revealed from the punctate colocalization pattern in distal dendrites (Figure 3B). In addition, preabsorption controls with GST-Muskelin 1–280, which had been used for antibody production, confirmed antibody specificity, as compared to a pre-immune serum control and anti-muskelin immunostaining (Figure 3C). Furthermore, upon expression of myc-muskelin in HEK293 cells, both anti-myc and anti-muskelin specifically detected a band of the same mass that was not detectable in mock-transfected control cells (Figure 3D). Together, we conclude that the newly generated antibody detects muskelin with high specificity.

**Figure 4:**
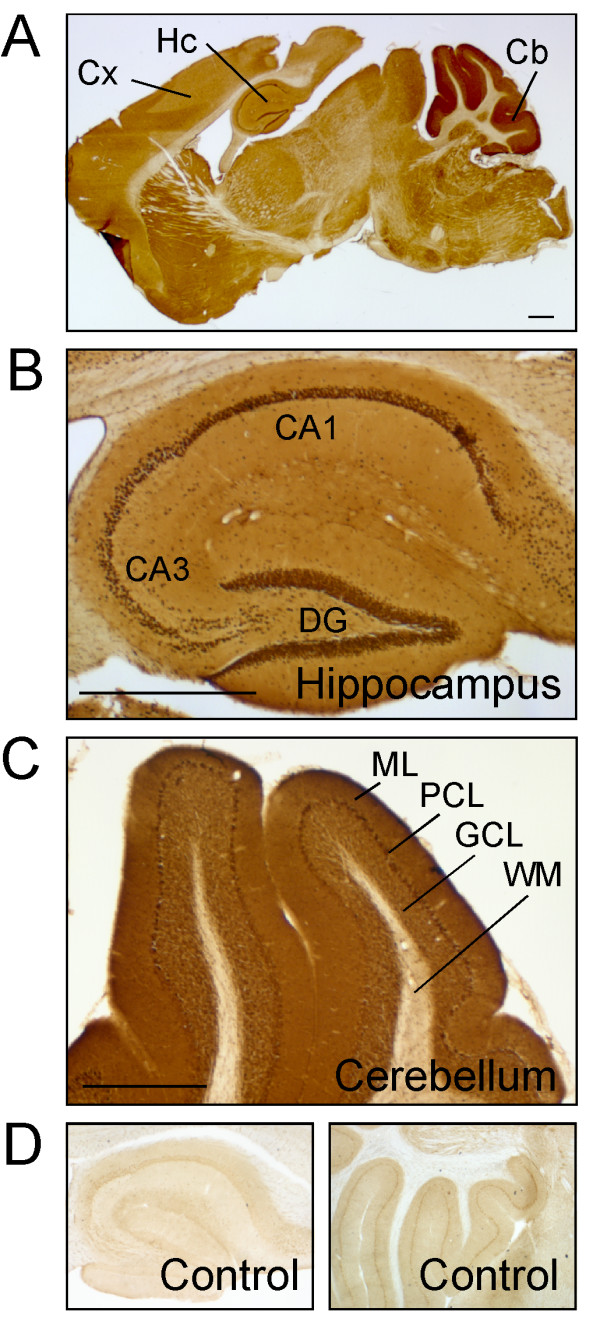
Tissue distribution of muskelin protein in brain sections of adult mice. (A) Whole brain section of an adult mouse brain. Muskelin displays widespread protein expression with high levels in hippocampus and cerebellum. (B) Within the hippocampus, muskelin-positive cells are found in CA1, CA3 and dentate gyrus regions. (C) Muskelin polypeptides are strongly expressed in cerebellar folia excluding the white matter. (D) Preimmune serum controls represent unspecific signals of the serum and confirm the specificity of signals in A-C. Key: CA1, Hippocampal CA1 region; CA3, Hippocampal CA3 region; Cb, Cerebellum; Cx, Cortex; DG, Dentate gyrus; GCL, Granule cell layer; Hc, Hippocampus; ML, Molecular layer; PCL, Purkinje cell layer; WM, White matter. Scale bars: (A-C) 1 mm.

**Figure 5:**
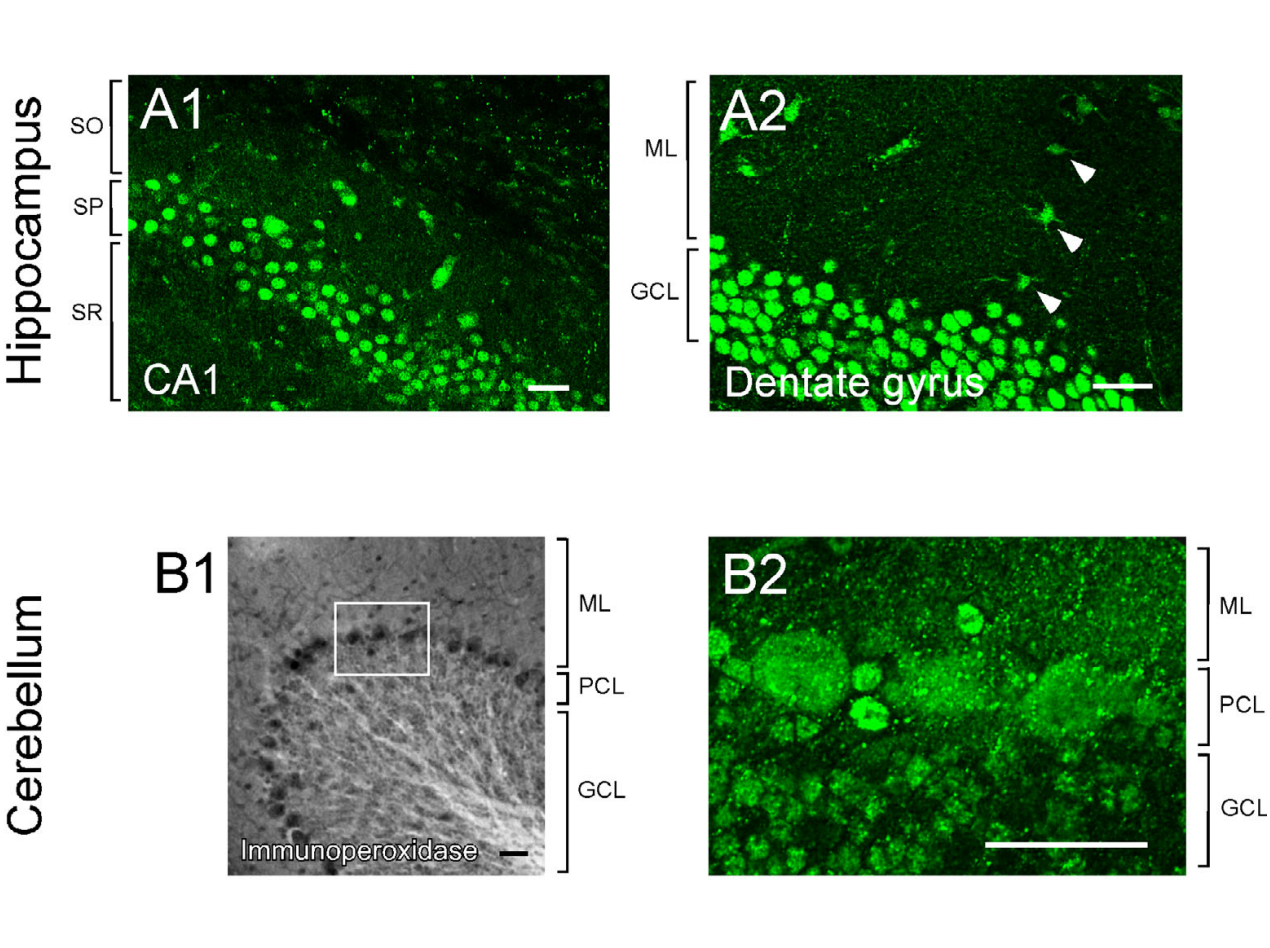
Muskelin is localized in nuclei, cell somata and neurite processes. (A1) Muskelin immunoreactive signals are strong in stratum pyramidale (SP) of the hippocampus containing cell bodies of CA1 pyramidal cells. Note that muskelin displays also punctate localizations in stratum oriens (SO) and stratum radiatum (SR), containing basal and apical dendrites of pyramidal cells, respectively. (A2) Muskelin immunoreactive signals are strong in the dentate gyrus granule cell layer (GCL) containing cell bodies of granule cells. A punctate muskelin localization is also found in the molecular layer (ML) harbouring granule cell neurite processes. Note, that also cells in the molecular layer, most likely representing interneurons (arrowheads), express muskelin. (B1, B2) In cerebellum, prominent staining is found in the Purkinje cell layer (PCL). High power magnification reveals that muskelin puncta are detectable in nuclei and cell bodies of Purkinje cells located in the Purkinje cell layer (PCL), as well as in the molecular layer (ML) harbouring Purkinje cell dendrites and parallel fiber axons of granule cells. Also in the granule cell layer (GCL) muskelin localizes in a punctate distribution in cell somata and neurites. Scale bars: 50 μm.

Immunoperoxidase staining of mouse brain sections revealed that muskelin polypeptides are expressed throughout the postnatal brain (Figure 4). Similarly, the highest protein expression was found in the hippocampus and cerebellum using the muskelin-specific antiserum, but was not detected by pre-immune serum control stainings. At high power magnification it was obvious that muskelin polypeptides are most prominent in hippocampal pyramidal cells of the *stratum pyramidale *and are also highly expressed in cells of the dentate gyrus granule cell layer (Figure 5A). Besides the strong immunoreactivity in nuclei, muskelin also displayed a punctate distribution in *stratum oriens *and *stratum radiatum*, which harbour basal and apical dendrites of pyramidal cells, respectively (Figure 5A1). Similarly, muskelin puncta were found in the molecular and polymorphic layers of the dentate gyrus. Individual cells in the dentate gyrus molecular layer, most likely interneurons, also expressed muskelin (Figure 5A2). Muskelin expression in the cerebellum could be detected in nuclei and cell bodies of granule and Purkinje cells with a punctate cluster formation in the molecular layer harbouring Purkinje cell dendrites and parallel fiber axons of granule cells (Figure 5B). We cannot exclude at this point that muskelin is also expressed in glial cells, however the majority of immunoreactive signal is derived from neurons. Together, these data suggest that muskelin localizes to distinct subcellular domains within neurites of mature neurons.

### Subcellular localization of muskelin in cultured hippocampal neurons

**Figure 6:**
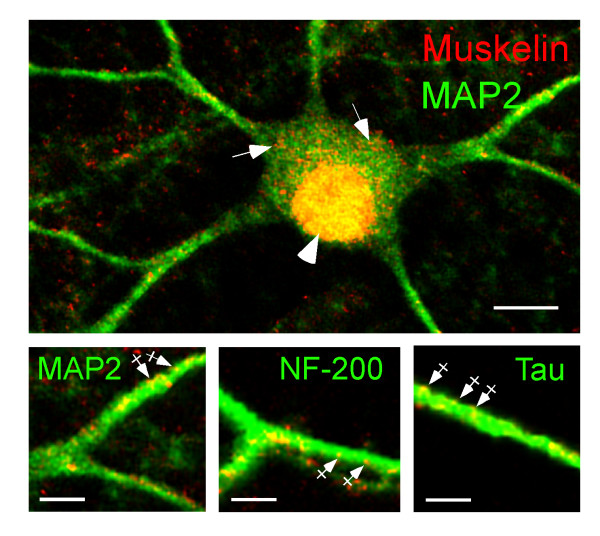
Muskelin localization at the subcellular level. Cultured hippocampal neurons were counterstained for muskelin (red) and individual markers (green). Muskelin localizes to the neuronal nucleus (arrowhead, yellow) and displays a punctate distribution in the cell soma (arrows) and in MAP2-positive dendrites (green) as well as in NF-200-positive or tau-positive axons (green). Muskelin immunoreactivity is often found at the margins of neurites (crossed arrows in magnified images below), suggesting surface membrane association. Overlay of green and red fluorescent signals are represented in yellow. Scale bars: 15 μm (top), 7 μm (bottom).

A punctate formation of muskelin immunoreactivity has been previously reported in non-neuronal cells and is suggested to result from head-to-tail interactions of muskelin molecules [5,13]. To address whether muskelin immunoreactive puncta localize to axons and/or dendrites, we performed co-immunostaining with respective marker proteins on cultured hippocampal neurons. Double detection of muskelin and microtubule-associated protein 2 (MAP2), a marker for neuronal dendrites [17], revealed muskelin puncta in both proximal and distal dendrites of neuronal cells (Figure 6). Furthermore, co-staining of muskelin with the axonal marker protein neurofilament-200 (NF-200) [18] or tau confirmed that muskelin puncta also localize to axonal projections (Figure 6). Notably, muskelin was often found at the margins of dendrites and axons, suggesting a close association with the plasma membrane (crossed arrows). Within cell bodies, large numbers of small muskelin puncta were detectable (arrows) and, consistent with muskelin interacting with RanBPM [12] a nucleocytoplasmic protein [19-21], significant amounts of muskelin immunoreactivity localized to neuronal nuclei (Figure 6, arrowhead).

**Figure 7:**
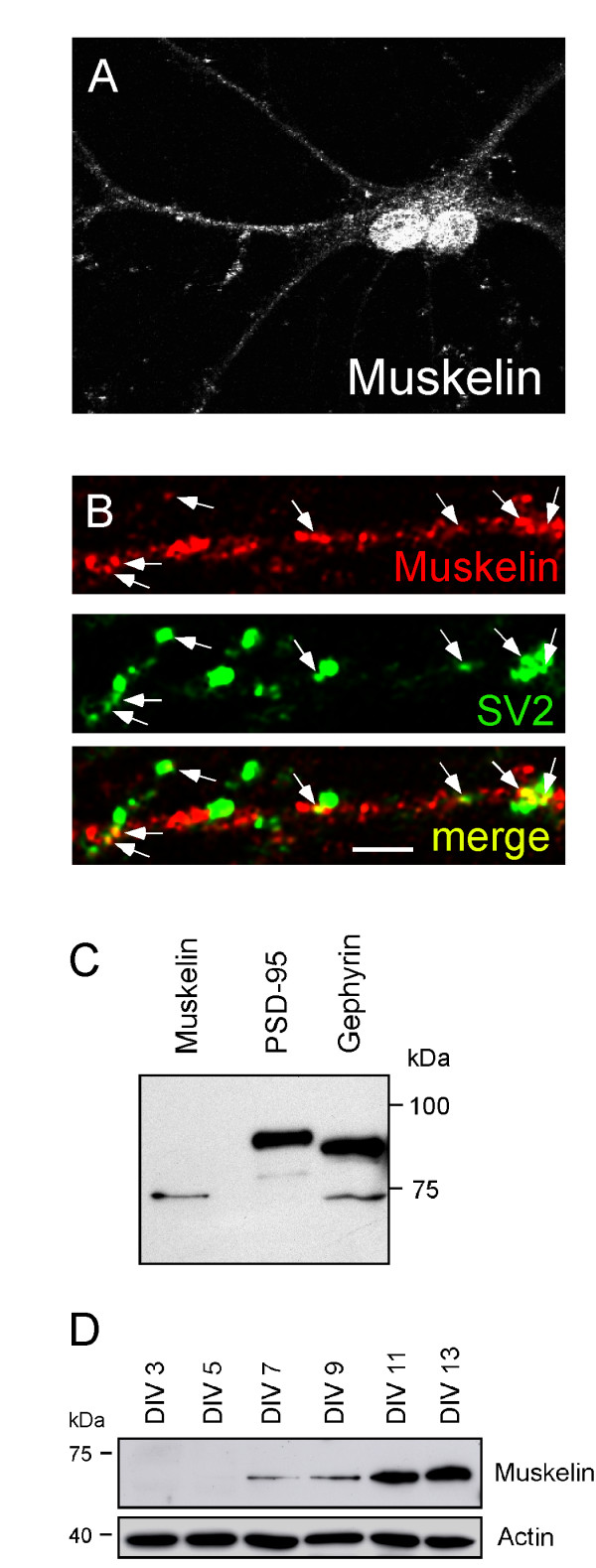
Muskelin puncta in dendrites partially colocalize with SV2-positive presynaptic axon terminals. (A) Detection of endogenous muskelin in cultured hippocampal neurons. Two neighbouring cells display prominent immunoreactivity in the nuclei and immunoreactive puncta in dendrites. (B) Co-staining of endogenous muskelin (red) and synaptic vesicle protein SV2 (green). Yellow puncta represent apposition of muskelin and SV2-positive signals (arrows) indicative for synaptic localization of muskelin. Scale bars: 20 μm (A); 3 μm (B). (C) Western blot analysis of synaptosomes prepared from P10 rats. Detection of the synaptic markers PSD-95 and gephyrin confirms enrichment of excitatory and inhibitory synapses, respectively. Muskelin is detectable within synaptosomal fractions. (D) Detection of muskelin expression in cultured hippocampal neurons from different developmental stages (DIV3-DIV13). Actin detection serves as a loading control.

In the context of the hypothesis that muskelin binding partners play functional roles in synaptogenic processes, we aimed to understand whether dendritic muskelin puncta (Figure 7A) localize to synapses. Costaining of muskelin and the synaptic marker SV2, which labels both excitatory and inhibitory presynaptic terminal boutons [22], revealed that the majority of muskelin puncta locate at non-synaptic positions, however individual puncta were clearly associated with SV2-positive sites, as judged from the precise apposition of fluorescent signals (Figure 7B, arrows). Moreover, preparations of synaptosomes from P10 rats that were positive for the postsynaptic marker proteins PSD-95 and gephyrin, which represent excitatory and inhibitory synapses, respectively, revealed the presence of muskelin at synapses (Figure 7C). A comparison of muskelin in cultured hippocampal neurons at different developmental stages (DIV3-DIV13) indicated that muskelin expression reaches detectable levels around DIV7 and increases until DIV13 (Figure 7D), a time window when the majority of synaptic contacts in cultured neurons form and mature [17,23]. Together, these data suggest that muskelin can in principle locate at synaptic membrane specializations and are consistent with the synaptic role of muskelin's direct binding partner p39 [8].

### Subcellular distribution of muskelin upon differential centrifugation of whole brain extracts

**Figure 8:**
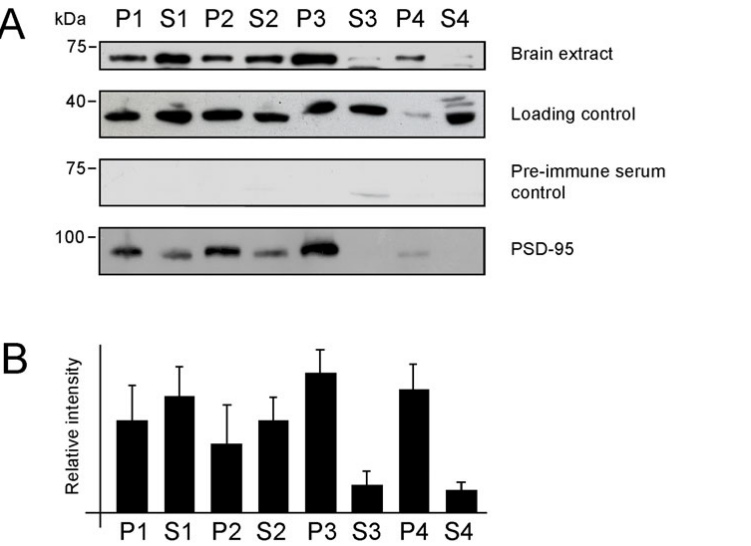
Muskelin distribution in whole brain cell extracts upon differential centrifugation. (A) Full length muskelin is abundant in the nuclear and cell remnant fraction (P1), the plasma membrane fraction (P2) and the respective supernatants (S1 and S2), which contain remaining membranes and the cytoplasm. Further separation reveals that relatively high amounts of muskelin are found in fraction P3, containing vesicular membranes and in fraction P4 that represents small vesicles and large protein complexes. In contrast, the soluble fractions S3 and S4 contain little muskelin. As controls, blots were detected against actin (loading control), with preimmune serum (antibody-specificity control) and with PSD-95, a protein known to be enriched at postsynaptic sites (P2) and at intracellular transport vesicles (P3). (B) The amounts of muskelin per fraction were quantitatively evaluated from three independent experiments using the NIH image software. For relative intensities, data were normalized against the respective actin signal, representing the relative amount of protein per lane. P: pellet; S: supernatant.

In non-neuronal cells, muskelin has been reported to distribute to membranes as well as to the cytosol [1,13]. To clarify whether brain muskelin co-fractionates with membrane structures, we undertook differential centrifugation of adult rodent whole brain extract. Western blot analysis of individual cell fractions revealed that the signal representing full-length muskelin was present in P1 pellets that contain nuclei and remaining intact cells (Figure 8A, B). Furthermore, P2 pellets, representing the plasma membrane fraction [24], and P3 pellets, consisting of small membranes and large vesicular structures [24,25], contained prominent amounts of muskelin. Notably, muskelin was found in P4 pellets representing small vesicles and large protein complexes, however was barely detectable in the remaining cytoplasmic fractions S3 and S4. Specificity of the antiserum used for analysis was controlled in Western blot experiments using pre-immune serum (Figure 8A). We also used PSD-95 detection as a control, since this protein has been previously analysed upon subcellular fractionation [24] and is known to be enriched at postsynaptic sites (P2) [26] and at intracellular cargo vesicles (P3) [27]. Together, these data suggest that the distinct subcellular localization of muskelin in neurons is due to associations with either membranes and/or intracellular protein complexes.

## Discussion

We have analysed the expression of muskelin in brain. Our main findings are: i), muskelin is expressed throughout the central nervous system with highest expression in the adult hippocampus and cerebellum; ii), muskelin localizes to the neuronal nucleus as well as to axonal and dendritic projections, including synaptic sites; iii), muskelin cofractionates with both membrane-enriched fractions and fractions containing protein complexes.

Muskelin was identified as a molecule required in cellular responses to TSP-1. Consistently, during mouse embryonic development, muskelin expression overlaps with the expression pattern of TSP-1 in brain [28]. However, transcripts of TSP-1 and of other TSP family members are strongly downregulated during the late phases of development and early adulthood [28-31], whereas widespread muskelin expression persists. In contrast, TSP-2 was found to be expressed in adult cerebellar Purkinje cells [32] and TSP-4 was detected in both the adult hippocampus and cerebellum [16]. These observations suggest that, besides a functional interaction of muskelin with TSP-1, muskelin might mediate other roles in neurons, independent of TSP-1 or other TSPs. For instance, the reported muskelin binding partner p39 in combination with its molecular target Cdk5, are candidate proteins that potentially impact on synaptogenesis by promoting rates of synapse formation [8-10].

In accordance with a possible role at neuronal membrane specializations, [6] our muskelin-specific antibody detected muskelin in a punctate distribution in cell somata, axons and dendrites, many of which were close to the plasma membrane. Confirming this observation, differential centrifugation revealed the presence of muskelin in membrane fractions. Counterstaining with a synapse marker and analysis of synaptosomes showed that a fraction of muskelin associates with synaptic contacts, suggesting that muskelin might participate in synaptogenic actions [4] and/or the p39/Cdk5 system [6,8,11].

Besides a widespread distribution of muskelin puncta in cell somata, axons and dendrites, the protein displayed prominent localization to neuronal nuclei. Interaction of muskelin with RanBPM [12], a polypeptide that locates to both the nucleus and the cytoplasm [19-21] is in agreement with this observation. Whether muskelin, which is present in both the cytoplasmic and nuclear compartments, is subject of nucleocytoplasmic transport is currently unknown. However, a role for muskelin in transport reactions is not unlikely, as the protein contains the LisH and CTLH domains, that in certain other proteins are known to interact with molecular motors of the dynein family [33], a microtubule-dependent recruitment system.

## Conclusion

In summary, our study provides the first comprehensive spatio-temporal analysis of muskelin expression in neuronal tissue and at the subcellular level in neurons. It reports a newly generated muskelin-specific antibody suitable for immunostaining and western blotting and indicates highest muskelin expression levels in hippocampus and cerebellum. At the subcellular level, muskelin is identified in a punctate distribution throughout the neuron, including synaptic sites, and also displays nuclear localization. Upon cell fractionation, muskelin is further enriched in fractions containing membranes and large protein complexes. The presented data generate an important point of departure to initiate functional studies in neurons in order to understand the role of a protein that appears to be at a critical interface between binding partners that are functionally involved in synaptogenesis, transport and nuclear processes.

## Methods

### *In situ *hybridization

Radioactively labeled RNA probes were prepared using the MAXIscript *in vitro *transcription kit (Ambion, Austin, TX) in the presence of α^35^S-UTP and α^35^S-CTP. Probes were purified with ProbeQuant sephadex G-50 microcolumns (Amersham, Buckinghamshire, England). *In situ *hybridization was performed as previously described [34-36], using five independent muskelin sequences: probe 1 (1–438 bp), probe 2 (450–885 bp), probe 3 (1151–1576 bp), probe 4 (1601–2024 bp) and probe 5 (1–1960 bp). 4 independent experiments with two sets of animals for each timepoint were used for each probe 1–4 (Figure 1B). 3 independent experiments with three sets of animals for each time point were used for probe 5 (Figure 1C).

### Antibody production

The N-terminal 280 amino acids of rat muskelin were cloned as an *EcoRI*/*SalI *fragment into pGEX-5X1 (Amersham, Buckinghamshire, England) (GST-Muskelin 1–280) and expressed in *E. coli *BL21. The fusion protein was bound to glutathione-agarose (Amersham, Buckinghamshire, England) and eluted overnight in the cold with 25 mM reduced glutathione (Sigma, Taufkirchen, Germany). The eluate was injected into a guinea pig with boosts every 30 days. The final bleed was taken after the third boost and the whole serum was used for Western blotting. For immunohistochemistry and immunocytochemistry, the immunoglobulins were affinity purified, using the immunogenic GST-fusion immobilized on a Hybond P membrane (Amersham, Buckinghamshire, England). Immunoglobulins were eluted with 250 mM glycine, pH 2.0 for 1 min, buffered with Tris-HCl, pH 8.0, aliquoted and frozen in liquid nitrogen. Preimmune serum was processed under identical conditions. For western blot controls, anti-myc (Sigma) was used at a dilution of 1:4000.

### Immunohistochemistry/immunocytochemistry

Mice from postnatal stages were perfused with 4% paraformaldehyde through the heart and sagittal vibratome sections were cut from the fixed brain. Sections were permeabilized with 0.4% Triton X-100 (Merck, Darmstadt, Germany) for 10 minutes and blocked with PBS, containing 10% normal goat serum and 1% BSA, for 1 hour at room temperature. Cultured hippocampal neurons were prepared as previously described [37]. Primary antibodies were applied in PBS containing 3% normal goat serum and 1% BSA overnight in the cold, as previously described [38]. The following primary antibodies were used: anti-MAP2 (1:1000; Chemicon, Hampshire, UK); anti-NF-200 (1:300; Sigma); anti-tau (1:5000, DAKO, Glostrup, Denmark); anti-SV2 (1:100; University of Iowa). Application of secondary antibodies and DAB development was carried out according to standard techniques. All secondary antibodies were from Dianova (Hamburg, Germany). For preabsorption controls, muskelin antibodies were incubated with GST-Muskelin 1–280. Antibody-antigen complexes were removed via pulldown with sepharose beads. Supernatant was adjusted to pH 8.0 with Tris-HCl and used for immunochemistry, as described. Fluorescence imaging was carried out with an inverted Leica TCS-SP2 laser scanning confocal microscope (Leica). For simultaneous multichannel fluorescence, images were taken in a sequential channel recording mode.

### Neuronal extracts and differential centrifugation

Buffers were supplemented with Complete Mini Protease inhibitor cocktail (Roche, Mannheim, Germany) with or without 1 mM PMSF. Differential centrifugation was performed as previously described [37]. In brief, P10 rats were decapitated and the brains put into buffer 1 (320 mM sucrose, 10 mM HEPES/KOH, pH 7,9, 1 mM DTT, 1 mM EGTA, 1 mM EDTA) and pottered using a glass-teflon-douncer. Extracts were centrifuged at 1000 × *g*, P1, and the supernatant, S1, further centrifuged at 10,000 × *g*, P2. The remaining supernatant, S2, was centrifuged at 100,000 × *g*, P3, with the supernatant referred to as S3. Supernatant S3 was again centrifuged at 400,000 × g, P4, with the final supernatant referred to as S4. Protein content was determined using a BCA protein assay kit (Pierce, Bonn, Germany). For SDS-PAGE, 30 μg of proteins were loaded in each lane. For Western blotting, the antiserum to muskelin was diluted 1:3000 and incubated over night in 5% milk in TBST (10 mM Tris, pH 8.0, 150 mM NaCl, 0.05% Triton-X-100). The preimmuneserum was applied under identical conditions. For controls, anti-actin (Sigma) was used at a dilution of 1:2000 and anti-PSD-95 (BD Biosciences, San Jose, CA) was used at a dilution of 1:250. For neuronal extracts, cultured neurons were prepared as previously described [37,38]. Cells were harvested in lysis buffer (PBS, 1% Triton-X-100) supplemented with Complete Mini Protease inhibitor cocktail (Roche, Mannheim, Germany) for 30 min on ice. Lysates were centrifuged at 1,000 × g for 5 min at 4°C. Protein concentration was determined using a BCA assay kit (Pierce, Bonn, Germany). Subsequently, protein samples were subjected to SDS PAGE and Western blotting.

### Preparation of synaptosomes

Buffers were supplemented with Complete Mini Protease inhibitor cocktail (Roche, Mannheim, Germany) and 1 mM PMSF. Brains of P10 rats were homogenized in sucrose buffer (320 mM sucrose, 1 M NaHCO_3_, 1 M MgCl_2_, 500 mM CaCl_2_, 500 mM EDTA, pH8.0) at 4°C and centrifuged at 1400 × g for 10 min. Pellets were resolved in the same ice cold buffer, homogenized and centrifuged at 700 × g for 10 min at 4°C. Subsequently, supernatants were centrifuged at 13,800 × g for 10 min at 4°C. At this step, pellets were resolved in 2 ml sucrose solution (320 mM sucrose, 1 M NaHCO_3_) and separated on a discontinous 1.2 M-0.85 M sucrose gradient for 2 h at 82,500 × g at 4°C. Synaptosomal fractions were recovered from the gradient at molarity 1–1.2. For Western blotting, the following antibodies were used: anti-muskelin (1:3000); anti-PSD-95 (1:250; Invitrogen/Zymed Laboratories); anti-gephyrin (BD Biosciences, 1:250).

## Abbreviations

The abbreviations used are: CNS, central nervous system; TSP1 thrombospondin 1; ECM extracellular matrix; Cdk5, cyclin-dependent kinase 5; LisH, lissencephaly-1 homology, CTLH, C-terminal to LisH; GST, glutathione-S-transferase; PMSF, phenylmethanesulfonylfluoride; GFP, green fluorescent protein; PSD-95, postsynaptic density protein of 95 kDa; SV2, synaptic vesicle protein 2.

## Authors' contributions

NT and SF performed *in situ *hybridization using four individual probes (Fig 1A, B and 2). ADA, EGL and JCA performed *in situ *hybridization using probe 1–1960 bp (Fig 1C). SL generated the muskelin-specific antibody. SL and NT performed immunohistochemical and immunocytochemical stainings. YP performed antibody controls and the synaptosome experiment. FH performed differential centrifugation and western blotting. MK designed and coordinated the study and wrote the manuscript with input from JCA. All authors read and approved the final manuscript.
